# Quickness of Pulse after Fever

**Published:** 1855-05

**Authors:** 


### QUICKNESS OF THE PULSE AFTER FEVER.

BY DR. STOKES.

"There is a case in the small fever ward to which I would wish to direct your attention. Although the patient has convalesced after a long fever, and is now gaining flesh and strength, we have found that the pulse continues rapid. Now, this is a circumstance which must always excite suspicion. In this patient, the signs of abdominal and pulmonary lesion have disappeared, as well as the characteristic expression of what may be termed the condition or state of fever,—yet, we find that his pulse does not correspond with the signs of improvement in all the other functions. It was suggested by Laennec, that the rapidity of pulse in patients after fever might depend on softening of the heart; but we shall see, by and by, that the true typhous softening of the heart, so far from inducing rapidity of pulse during convalescence, has much more frequently the effect of making it slow; not only slow as considered with reference to the condition of health, but actually falling below the ordinary standard. * * * * *

"These cases of quickness of pulse are of two kinds. In one class the pulse has never lost the rapidity it attained during the fever; or it has, perhaps, come down fifteen or twenty beats in a minute, and its rate then remains stationary. In the other cases the pulse, which had become quiet, rises to 100 or 120, or even higher, and remains at that rate for days together, without our being able to detect any cause for its increased rapidity. This, I think, is the worst case of the two; at least, it appears more often to indicate a new pathological change.

"The local diseases which have been found most frequently to attend this condition are of two kinds; one of them is tuberculosis

—the deposition of tubercles in the lung and other parts; the other is the existence of a secondary reactive inflammation in the mucous glands of the intestines. To this subject Dr. Cheyne long ago drew attention, in speaking of imperfect convalescence in typhus fever; and he gives several cases in the Report of the Hardwicke and Whitworth Hospitals, in which patients had recovered well from typhus fever; had, to all appearance, regained a certain degree of strength; had regained their appetite: but they showed no disposition to leave their beds; the pulse gradually got quicker and quicker; the belly swelled; diarrhœa came on; and the patients died with symptoms of disease of the intestinal canal. Upon dissection, extensive ulceration of the mucous glands was found in the intestine. These are the two most common of the local diseases which you should suspect when you have a patient who has gone through a long fever with the pulse continuing or becoming very quick.

"But now suppose that you examine such a patient with great care. You percuss his chest; you examine the state of his respiration in every way, and you cannot satisfy yourself that there is any disease in his lung; and you will recollect what I mentioned to you at our last lecture, that in most cases of this tuberculosis after typhus there is great constitutional suffering. Well, you may make up your mind, from the absence of all these signs, that the patient is not becoming tuberculous, at all events. When you proceed to examine the abdomen, you will find, perhaps, that he has a good appetite; that his thirst is gone; that the belly is hollow and soft, and there is no tumefaction of it; that there is no tenderness on pressure anywhere; no throbbing of the abdominal aorta; no tendency to diarrhœa—in fact no one symptom of disease of the mucous membrane of the intestine. And yet, as in the case above stairs, you have a pulse with this unpleasant degree of quickness. I rather think that this man's pulse is now quicker than it was on the twenty-first day of his illness; and it makes me extremely uneasy about him. Now, gentlemen, suppose that you did not find either disease of the lung or disease of the abdomen; what should you suspect? Generally, in those circumstances, you may suspect that the patient will be attacked by phlegmasia dolens; for we have seen a considerable number of cases in which, after fever, where the pulse continued rapid, this disease exploded. It is, I think, more likely to occur in the non-petechial than in the petechial cases; it is more likely to occur in the long fevers than in the short fevers; it is very liable to arise in patients who have had a fever running on beyond twenty-one days, or thirty days, or forty days. In these patients, after the true symptoms of fever have subsided, they remain with a rapid pulse, and probably, in a week or ten days, symptoms of phlegmasia dolens come on; and the disposition to this venous inflammation is very curious in them, for you will very often find that the patient has two or three distinct attacks of it. It may affect one leg, and you will get the patient through that attack; still the pulse does not regain its natural rate. After a week or ten days, the other extremity will be attacked: and it is even possible that a third seizure may occur, as it were, a relapse of the disease in the part first affected; and in this way patients will go on laboring under these attacks and their consequences for months together. In most instances, however, the patients recover. In most of the cases I have seen, of this acute phlebitis consequent upon fever, there was distinct notice of the invasion of the disease—that is to say, the patient was attacked with pain in the calf of the leg. He is attacked, say in the course of the night, with pain in the calf of the leg; and when you come, in the morning, you find him exhibiting all the characteristics of the disease—a large swelling, pain on pressure, and all the other symptoms. Sometimes you find a cordy state of the superficial veins; at other times not. When you can feel a deep-seated vein, you will sometimes find it in a hard and cordy state.

"I think it right to warn you of these curious circumstances; for I am sure that in the course of your practice, you will often be in this position, that you will have a patient recovering from fever, and going on in every respect well, except that the pulse does not come down. The rule then, is, that if the most minute examination fails to detect disease in the great viscera, you may expect the occurrence of phlebitis.

"The term, phlegmasia dolens, under these circumstances, is not always applied correctly; for the disease is not always painful. We have seen a few instances in which the discovery of the local affection was entirely accidental. Of course, you will not suppose that I am prophesying that the patient above stairs will have phlegmasia dolens; all I say is, that he is in that state which would justify you in suspecting something of the kind.

"I have mentioned the rapid deposition of tubercle, ulceration of the intestines, and phlebitis of the extremities, as the diseases we have found to occur most commonly in these instances of unaccountable quickness of pulse after fever. Doubtless, there are many more examples of local disease arising under these circumstances; but the general rule will hold good, that this symptom foreshadows a disease, which, though at first latent, will before long become manifest. These diseases are generally attended with much irritation, and the condition of the patient is rather one of irritation, or inflammation if you will, than of essential fever. And this is one of the illustrations of a circumstance often observed by a clinical investigator, namely, the change of character of disease, locally and constitutionally, in the same patient, and within a not very extended period. The typhous condition, generally considered, will change into a different state. The essential or general morbid state will disappear, and a local irritation, with its symptomatic fever, becomes the prominent malady. Nay, you will sometimes find that the very condition of a local disease, formed during the first, the typhous or essential period of the disease, will itself change, and take on the characters of what is termed by some a 'healthy inflammation.' You may sometimes see this well illustrated in that terrible disease, accompanied by purulent deposits, in many of the articulations. The patient may throw off the typhoid state which attends the earlier periods of the disease, and then the affection of the joints seem to change in its nature, and take on the characters of ordinary arthritis. I have only seen this, however, where one or two of the larger joints had been affected with the primary disease; and it was most remarkable to witness the changes both in the constitutional state and the local affection. It was no longer necessary to use general stimulation; it was no longer improper to employ local antiphlogistic measures."—*Clinical Lectures on Fever*, No. 10.—*Ranking*.

